# Feasibility study of a smartphone app to monitor systemic sclerosis-related digital ulcers—a potential new tool for remote clinical monitoring

**DOI:** 10.1093/rap/rkag035

**Published:** 2026-03-18

**Authors:** Paul New, Graham Dinsdale, Andy Vail, Joanne Manning, Michael Hughes, William G Dixon, Chris Taylor, Mark Dickinson, Ariane L Herrick, Andrea Murray

**Affiliations:** Centre for Musculoskeletal Research, Division of Musculoskeletal and Dermatological Sciences, The University of Manchester, Manchester Academic Health Science Centre, Manchester, UK; Rheumatology Department, Salford Royal Hospital, Northern Care Alliance NHS Foundation Trust, Salford, UK; Centre for Biostatistics, The University of Manchester, Manchester Academic Health Science Centre, Manchester, UK; Rheumatology Department, Salford Royal Hospital, Northern Care Alliance NHS Foundation Trust, Salford, UK; Centre for Musculoskeletal Research, Division of Musculoskeletal and Dermatological Sciences, The University of Manchester, Manchester Academic Health Science Centre, Manchester, UK; Rheumatology Department, Salford Royal Hospital, Northern Care Alliance NHS Foundation Trust, Salford, UK; National Institute for Health and Care Research Manchester Biomedical Research Centre, Manchester, UK; Rheumatology Department, Salford Royal Hospital, Northern Care Alliance NHS Foundation Trust, Salford, UK; National Institute for Health and Care Research Manchester Biomedical Research Centre, Manchester, UK; Division of Informatics, Imaging and Data Sciences, The University of Manchester, Manchester, UK; School of Engineering and School of Health Sciences, The University of Manchester, Manchester, UK; Photon Science Institute, The University of Manchester, Manchester, UK; Centre for Musculoskeletal Research, Division of Musculoskeletal and Dermatological Sciences, The University of Manchester, Manchester Academic Health Science Centre, Manchester, UK; Rheumatology Department, Salford Royal Hospital, Northern Care Alliance NHS Foundation Trust, Salford, UK; National Institute for Health and Care Research Manchester Biomedical Research Centre, Manchester, UK; Centre for Musculoskeletal Research, Division of Musculoskeletal and Dermatological Sciences, The University of Manchester, Manchester Academic Health Science Centre, Manchester, UK; Photon Science Institute, The University of Manchester, Manchester, UK

**Keywords:** digital ulcers, scleroderma, smartphone app, smartphone photography, systemic sclerosis

## Abstract

**Objectives:**

To assess the feasibility, in a clinical setting, of using a smartphone app that allows ‘tracking’ of SSc-related digital ulcers/lesions over time and to gauge patient opinion of the app’s benefits, including a newly added lesion feedback function for patients.

**Methods:**

Patients with SSc with active digital ulcers/lesions were recruited and assessed twice: on study entry and at the point of lesion healing. Participants photographed their lesions and recorded patient-reported outcome measures (PROMs) with the app twice weekly. The lesion feedback function showed lesion size (measured from the photographs) over time. Ways in which feasibility was assessed included the number of photographs and PROMS recorded via the app, data from patient questionnaires and verbal feedback.

**Results:**

A total of 10 patients were recruited: 9 (90%) female, median age 55 years, median disease duration 12 years. Nine (90%) completed the study. The median duration of study participation per ‘lesion episode’ was 39 days (range 18–140). A total of 210 images were returned, with a median of 12 (range 4–87) per episode, and 99 sets of visual analogue scale/pain scores were returned, with a median of 7 (range 3–24) per episode. On a scale of 1 (very easy/helpful) to 10 (very difficult/not helpful), the median score for ease of sending photographs was 1 (range 1–3) and for helpfulness of feedback it was 1.5 (range 1–8). All patients were keen to use the app again.

**Conclusion:**

The feasibility and benefits of using the smartphone app, including the feedback function, lend support to possible wider use of the app in routine clinical practice.

Key messages

- Using a smartphone app to monitor finger lesions is feasible for most patients with SSc.
- Patients found the feedback function helpful and all were keen to use the app again.
- App-based remote monitoring of finger lesions may be useful in future routine clinical practice.

## Introduction

Painful, disabling digital ulcers develop in ≈50% of patients with SSc [1, 2] and can be slow and difficult to heal [3]. Clinical trials to identify new, effective treatments have proved challenging, in large part because of a paucity of reliable, objective outcome measures. It was against this background that we developed a smartphone app to allow ‘tracking’ of SSc-associated digital ulcers and their associated pain and disability via capture of a combination of photographs and patient-reported outcome measures (PROMs) [4]. This was with a view to developing an app-based outcome measure to facilitate clinical trials. App development was informed by a patient user group, who felt that the app might be helpful not only for clinical trials but also in routine clinical care, as this could provide a means of their clinician viewing the ulcer(s) and assessing treatment response remotely, thus reducing the need for hospital visits. Members of the user group also stated that they would value feedback about their ulcer(s) between hospital appointments.

In view of these comments from patients, the aims of this study were to assess the feasibility of using the previously developed app in a clinical setting (including patients’ perceptions regarding how easy/difficult it was to use the app in the ‘real world’) and to gauge patient opinion on the benefits of using the app, including a newly added feature of providing feedback to patients on ulcer/lesion size. It was outside of the scope of this feasibility study to assess the impact of using the app on patient care, or the effects of any intervention, and patients were aware that (in this feasibility study) information obtained via the app would not influence their clinical care. Because of the challenges in defining ‘digital ulcers’ [5–9], the broader term ‘digital lesions’ will be used from now on in this report.

## Methods

### Participants

Patients who fulfilled the 2013 classification criteria for SSc [10] or who had an SSc-spectrum disorder and who had one or more digital lesions were invited to participate. All were adults attending a single UK tertiary centre for SSc. Patients were recruited either face-to-face (usually when attending an outpatient appointment) or remotely. The study was approved by the South Central–Oxford A Research Ethics Committee and all patients signed informed consent (reference: 23/SC/0214). A patient could be re-recruited if they developed a new lesion after initial study completion.

### Patient pathway through the study

Participants had two study visits, one at the beginning of the study and one at the end, which was at the point of lesion healing. At the first visit, patients downloaded the app (of which a detailed description is given in Davison *et al.* [4]) onto their own smartphone and were trained in the use of the app (see supplementary material for the imaging protocol). Patients without a smartphone were given the option of being loaned one. They were asked to complete a ‘smartphone usage’ questionnaire to gauge their familiarity with smartphone technology. Additional technical support via telephone call was available to participants throughout the study.

**Figure 1:**
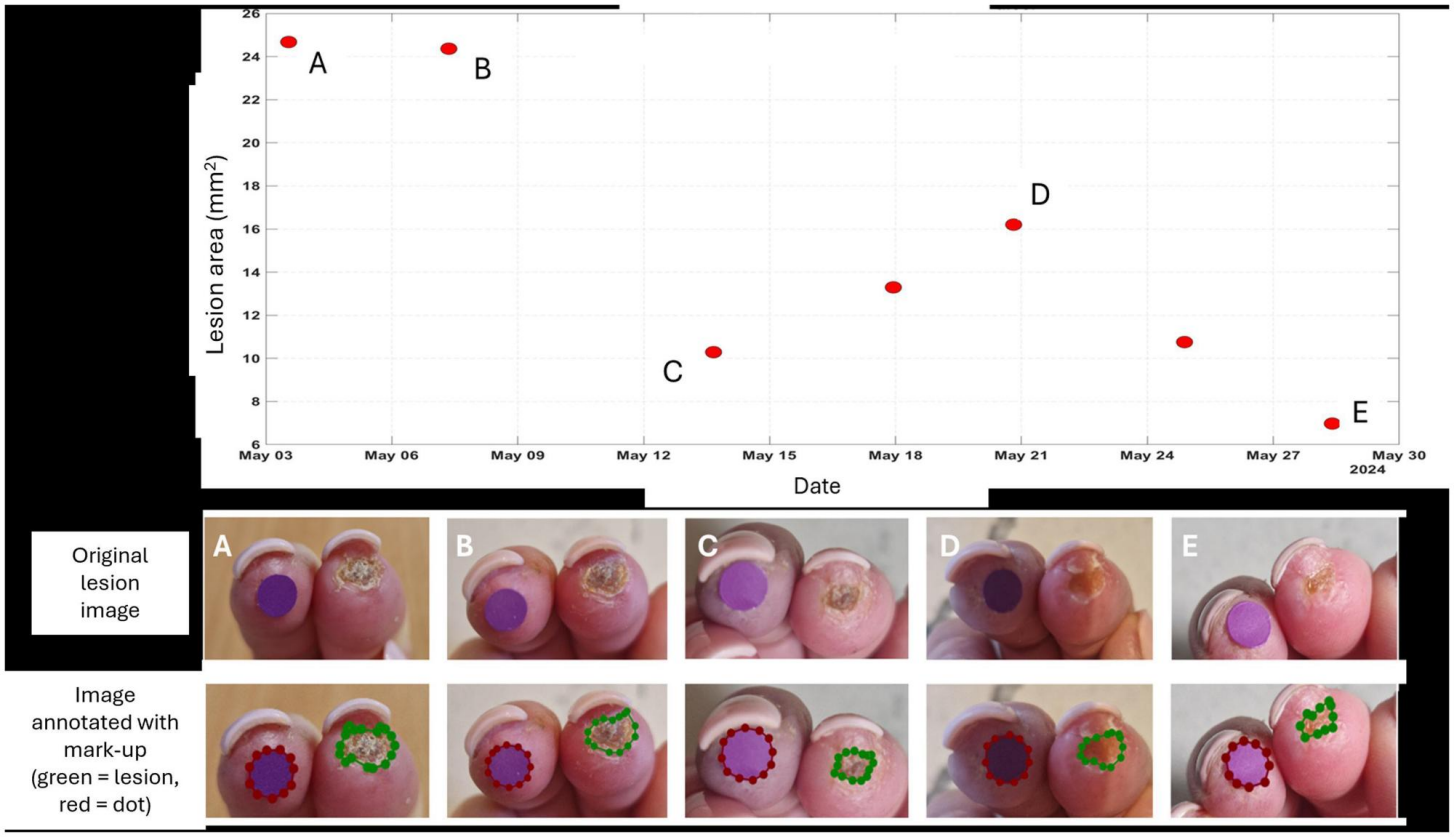
An example of digital lesion healing over time. Top: example of a lesion ‘feedback’ graph sent to participants during the study. The lesion size on the vertical axis is plotted against the date/time stamp of the image from which it was derived (A–E relate to the lesion images below, letters not present when viewed by patients in the study). Bottom: A–E, example of one lesion over time, top row, original image, bottom row showing the markup of the boundary (green), used to extract lesion size for feedback. The purple ‘dot’ sticker (6 mm diameter, placed on adjacent finger to lesion) was used to calibrate the size of the lesion in the images

Patients then imaged their lesions and recorded PROMs with the app twice weekly until the point at which the patient perceived the finger lesion was healed. The study duration therefore differed between patients, in contrast to our previous study which was for 30 days only [4]. User group discussions suggested that people would prefer to collect photographs and PROMs less often than the daily assessments of our previous study [4]. The specific data collected were similar to our original study [4] and in summary were:

- Lesion photographs. Lesion size was measured using a calibrated marker on the photographs (Fig. 1).
- Pain score, as assessed by a Likert scale of 0 to 10 (10 = worst possible pain).
- Three visual analogue scales (VASs) for interference of daily activities by Raynaud’s phenomenon (Raynaud’s-VAS) or by finger ulcers/lesions (Finger lesion-VAS) and overall disease severity [Disease severity-VAS; scale 0–100, completed using a ‘slider’ (100=very severe limitation)]. These VAS scales were modified from the VAS of the Scleroderma Health Assessment Questionnaire [11].

In addition to this twice-weekly data collection, participants were also asked to complete the Hand Disability in Systemic Sclerosis—Digital Ulcers (HDISS-DU) questionnaire [12] at visit 1, every 4 weeks thereafter and at the final study visit.

### Feedback to patients

A new feature added to the app specifically for this study was the ‘feedback’ function. A graph showing lesion size over time was made available to users in the app. An example is shown in Fig. 1.

### Assessing feasibility

Feasibility was assessed in different ways. First, by the number of eligible patients who, having read the patient information sheet, elected to participate. Second, by the number of photographs and PROMs recorded via the app and over how many days. Third, from opinion obtained from a feedback questionnaire (which can be viewed in the supplementary material) completed by all patients on study completion: questions included how easy it was to send photographs and data via the app to the research team. Fourth, by verbal feedback at a patient user group held on 9 December 24, towards the end of the study.

### Assessing patient perception on benefits of using the app

This was assessed via the feedback questionnaire and also at the user group meeting towards the end of the study.

## Results

Of the 22 patients with digital lesions who were sent a patient information leaflet, 10 were recruited into the study, with one consenting for two ‘episodes’ (therefore 11 episodes in total) (Supplementary Fig. S1). The patient who consented for two ‘episodes’ completed an end-of-study questionnaire on each occasion (both have been included in the analysis). One patient withdrew because of ill health after submitting only one photograph. Of the 10 completed episodes, 3 included two lesions, with evaluable photographs from 13 lesions (Supplementary Fig. S1). The median duration of study participation per lesion episode was 39 days (range 18–140). During study participation, all 10 patients received oral vasodilator therapy (none had admissions for intravenous iloprost therapy), 1 underwent surgical debridement and 4 had one or more courses of antibiotic therapy.

All 10 participants fulfilled the ACR/EULAR criteria for SSc [10]. Nine (90%) were female, 7 (70%) had lcSSc and 3 (30%) had dcSSc. Their median age was 55 years (range 36–75), median duration of RP was 27 years (range 14–33) and median duration of SSc (from date of first non-RP manifestation) was 12 years (range 2–30). Five (50%) were ACA positive, 1 (10%) was anti Scl-70 positive and 2 (20%) were anti-RNA polymerase III positive. Eight (80%) were on vasodilator therapy and 3 (30%) were on bosentan.

The pre-study questionnaire showed that all participants owned a suitable smartphone, which they used for the study. Of the 13 digital lesions, 9 (69%) were on the dominant hand, 9 (69%) were located on the fingertip and 4 (31%) on an extensor surface. At baseline, median values for PROMs (10 participants) were as follows: pain score 5.5 (range 0–9), Raynaud’s-VAS 39.5 (range 0–100), Finger lesion-VAS 76.5 (range 0–100) and Disease severity-VAS 50 (range 0–100). The median score for the HDISS-DU questionnaire at baseline was 3.42 (range 1.00–4.33).

### Feasibility of using the app

#### Proportion of patients agreeing to participate

Of the 22 patients approached about the study, 4 (18%) were not interested, including 1 who was ‘too busy’ and 1 who was about to go on holiday (Supplementary Fig. S1). Of the other eight who did not participate, two were about to have surgery for the lesions and in six the lesion had almost healed.

#### Completeness of submitted images and PROMS

In total, 210 images were returned [median 12 (range 4–87) per episode] from the 10 lesion episodes. Ninety-nine sets of VAS and pain scores were returned out of a possible total of 144 (69%) [median 7 (range 3–24) VAS/pain scores per episode].

#### As assessed by post-study questionnaire

Responses to questions in the post-study questionnaire are described in Table 1 with responses on a 1–10 scale, including (1 = very easy or very keen, 10 = very difficult or not keen) ‘Overall experience of using the mobile phone to photograph/video your finger ulcer’ [median score 2.5 (range 1–10)] and ‘Would you be keen to use the app again (to monitor the progress of any further ulcers)’ [median score 1 (range 1–4)]. One participant required help from someone else to take the photographs and one patient used a tripod. Four patients reported difficulty in pressing the button or screen. The patient with the most difficulties reported ‘cracked skin’ and limited hand mobility.

**Table 1:** Responses to the post-study questionnaire from the 10 ‘episodes’ of the 9 participants who completed the study.

Question^a^	Median (range)
Remembering to take photos/videos of finger ulcers (twice per week)	3 (1–6)
Taking photographs/videos at the same time on each occasion (twice per week)	3 (1–6)
Taking photographs/videos in the same place on each occasion (twice per week)	2 (1–6)
Keeping the environment and lighting the same on each occasion (twice per week)	1.5 (1–5)
Making sure the hands were in the same condition (e.g. no recent hand cream or hand washing) on each occasion (twice per week)	1 (1–4)
Overall experience of using the mobile phone to photograph/video your finger ulcer	2.5 (1–10)
Holding the phone when imaging	4 (1–9)
Pressing the button or screen to take an image	4 (1–8)
Getting a good clear image of your finger ulcer	2 (1–7)
Was it helpful to know (via app feedback) whether your ulcer was improving (getting smaller) or worsening (getting bigger) over time?^b^	1.5 (1–8)
Was it easy to understand the feedback provided via the app (was it presented in a clear and concise manner)?	2 (1–8)
Would you be keen to use the app again (to monitor the progress of any further ulcers)?^c^	1 (1–4)
How easy was it to send images/video to the research team through the SALVE app?	1 (1–3)

a Answers on a 1–10 scale. 1 = very easy, 10 = very difficult unless otherwise stated.

b Answers on a 1–10 scale. 1 = very helpful, 10 = not helpful.

c Answers on a 1–10 scale. 1 = very keen, 10 = not keen.

#### As assessed at a patient user group

The four patients attending the final user group meeting towards the end of the study unanimously confirmed feasibility.

### Patient perception on benefits of using the app

#### As assessed by post-study questionnaire

All but one participant felt it was helpful to know if the lesion was improving or worsening [median score 1.5 (range 1–8) (Table 1). The participant who did not find it helpful commented that they knew if the lesion was changing. Most participants found it easy to understand the feedback and all would be keen to use the app again (Table 1). As a result of using the app, three participants stated that they would be less likely to seek medical advice, two who would be neither more nor less likely and three who would be more likely. The participant who completed two episodes (and two end-of-study questionnaires) initially stated neither more likely nor less likely, and subsequently more likely.

Five participants were happy submitting data from the app twice weekly, three would have preferred daily and one would have preferred weekly.

#### As assessed at a patient user group

In the final user group meeting there was discussion about how the lesion size data should be presented for accessibility and understanding. Participants suggested a graph for clinicians and a pictorial interpretation for patients, showing a relative change in lesion size. This could be either a circular area (presented as a ‘bull’s-eye’) or an area that showed the shape of the lesion (Supplementary Fig. S2). Patients also suggested that percentage change and ‘larger’ or ‘smaller’ would be helpful to interpret the longitudinal data.

## Discussion

This study confirms previous findings that using a smartphone app is feasible for most patients with SSc [4, 13]. This is despite the well-recognised problems with hand function in patients with SSc [14], evidenced in the 10 participants by their high levels of pain and by the impact of their disease on their everyday activities. Most participants submitted images and PROMs regularly, with very positive responses to using the app as gauged by both the feedback questionnaire and by discussion at the user group meeting. The key point was that most patients found the app very easy to use. The duration of app usage was longer than in our previous study, continuing to the point of lesion healing: one patient submitted images and PROMs for as long as 140 days, suggesting that this monitoring method is feasible even for lesions that are slow to heal.

A novel aspect of this study was the provision of feedback to patients regarding lesion size: most patients found this helpful to gauge the ‘progress’ of their lesion and the feedback could also be helpful for their clinicians. A strength of the study was the strong level of patient engagement via the user group and in the next iteration of the app we intend to incorporate the suggestion of including (in the feedback) percentage change in lesion size. Our study had limitations, including the small number of patients included, although 10 participants was an appropriate number to test the feedback function. Also, we did not assess clinician opinion regarding patient benefit in terms of whether use of the app reduced the number of hospital visits and/or whether use of the app influenced their clinical decisions (e.g. whether to prescribe an antibiotic). Clinician opinion will be factored into future work to allow more definite evaluation as to whether the app provides benefit in terms of both high-quality clinical care and cost-effectiveness.

Digital health technologies are gaining momentum in many branches of medicine, including (relevant to ulcer/lesion healing) diabetic foot ulcers [15]. Our data support further development of app-based remote monitoring of SSc-related finger lesions (and potentially of other chronic wounds) and larger, controlled studies to evaluate clinical impact and integration into routine care. Our expectation is that once patient-generated data are integrated into routine clinical care [16, 17], app usage may facilitate early identification of clinical deterioration and therefore early intervention, thus potentially reducing pain and morbidity, and (for many patients) the need for hospital attendance.

## Supplementary Material

rkag035_Supplementary_Data

## Data Availability

The sponsor will share de-identified individual participant data collected during the study with researchers who provide a methodologically sound proposal.
