# Supplementary material for: Feasibility study of a smartphone app to monitor systemic sclerosis-related digital ulcers—a potential new tool for remote clinical monitoring
Source: Rheumatol Adv Pract. 2026 Mar 18;10(2):rkag035. doi: 10.1093/rap/rkag035 (PMC13070691; doi:10.1093/rap/rkag035)
Supplement: rkag035_Supplementary_Data [file rkag035_supplementary_data.zip › PNew_21-1-26_Supplementary_Clean.docx]

FEASIBILITY STUDY OF A SMARTPHONE APP TO MONITOR SYSTEMIC SCLEROSIS-RELATED DIGITAL ULCERS - A POTENTIAL NEW TOOL FOR REMOTE CLINICAL MONITORING: SUPPLEMENTARY MATERIAL

Supplementary Figure S1 - Consort diagram

1 patient withdrawn.

- Due to ill health.

Too busy (n=2)

Health issues (n=3)

Ulcer virtually healed (n=1)

Not wish to take part (n=3)

- =1)
- Too busy (n=2)
- Health issues (n=3)
- Ulcer virtually healed (n=1)
- Not wish to take part (n=3)
- 8 consented for 1 episode.
- 1 consented for 2 episodes.

10 episodes completed.

2 patients (3 episodes) had 2 lesions. 13 lesions in total.

r virtually healed (n=1)

Not wish to take part (n=3)

- =1)
- Too busy (n=2)
- Health issues (n=3)
- Ulcer virtually healed (n=1)
- Not wish to take part (n=3)

Excluded / declined to take part (n=12)

- Ulcer/lesion virtually healed (n=6)
- Due to have surgery on lesion (n=2)
- Not interested in participating (n=4)

Too busy (n=2)

Health issues (n=3)

Ulcer virtually healed (n=1)

Not wish to take part (n=3)

- =1)
- Too busy (n=2)
- Health issues (n=3)
- Ulcer virtually healed (n=1)
- Not wish to take part (n=3)

Completed Study (n=9)

- 9 consented for 1 episode.
- 1 consented for 2 episodes.

11 episodes consented.

Health issues (n=3)

Ulcer virtually healed (n=1)

Not wish to take part (n=3)

- =1)
- Too busy (n=2)
- Health issues (n=3)
- Ulcer virtually healed (n=1)
- Not wish to take part (n=3)

Consented (n=10)

Assessed for eligibility (n=22)

Supplementary Figure S2. An example of a lesion healing with time; A) at baseline and B) later in time. C) Possible pictorial feedback that the patient user group suggested for patient feedback, representing the changing size of the lesion (left showing the shape of the lesion and right a more simple circle). It was suggested that the graph in Figure 1 (main manuscript) could be used for clinician feedback. It was also suggested that there could be accompanying text telling patients whether the lesion had increased or decreased in size and by what percentage.


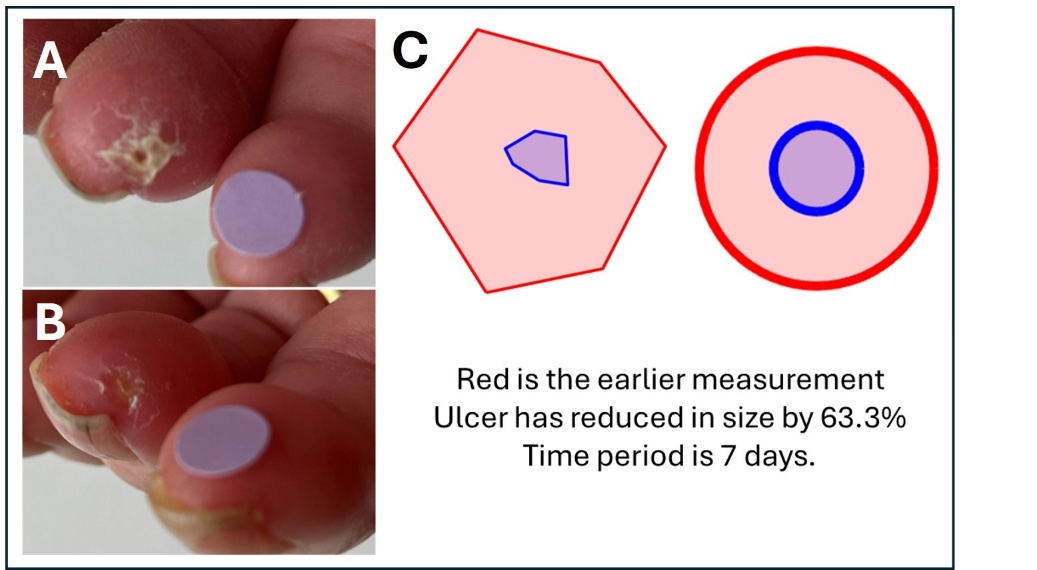


**Development of a measuring app for finger lesions as an outcome measure for systemic sclerosis-related digital ulceration**

**Digital lesions (finger ulcers) - Imaging Protocol**

**Objective:** A simple set of instructions for patients to follow to allow them to capture high-quality images (photographs and optionally videos) of their finger lesions using a smartphone camera and app.

**Scope:** For use by study participants testing a smartphone app as part of SALVE study 3 (Scleroderma App for Lesion Verification 3).

**Assumptions:**

1. Participant has a smartphone (their own or loaned) with built-in camera and has had the SALVE study app installed onto the device.
2. Participant has one or more finger lesions.

**Imaging, frequency, timing, and sizing reference:** Participants will be asked to image each finger lesion twice per week for the duration of the study. It will be explained to them that this should ideally occur at the same time (e.g. just before bedtime). An app notification will alert the user when the next image is due. Patients will be provided with sticky dots (circular, 6mm in diameter) to be placed on their skin, next to their lesions, to provide a sizing reference.

**Environment:** the photographs/videos should, where possible, be taken in the same location every day. This is likely to be in the participant’s own home and should be consistently well lit and as free from visual clutter as is possible. It is recommended that there is a flat surface, such as a table or countertop on which the participant can rest their hands while imaging – this is to reduce the possibility of motion artefacts (blur). Keeping the background of the image in high contrast to the hand of interest is very important to ensure no hand features are lost or hard to identify. If a countertop, or similar is used, it should not have features that could hinder analysis, such as household items or a woodgrain pattern.

**Considerations before imaging:** (to be discussed with the participant to take account of daily routines etc.) To help with consistency in imaging, it is best if the hands are in the same “state” at each imaging session. This should include time since last washed, application of hand creams or treatments, and removal or application of dressings on finger lesions. If dressings are not to be removed on a particular imaging day, then the participant can select “imaging not possible today” in the app, to skip the session.

**Taking photographs/videos:** Users have two options for photographing their finger lesions: (1) is to use the rear (standard) camera on the phone and photograph one hand while holding/operating the phone with the other; (2) is to use the front-facing camera (screen side/selfie camera) with the phone lying flat on a surface facing upwards. This second option is perhaps best for those participants with issues with hand function.

Participants can use a tripod stand if this makes it easier for them to image their finger lesions, or if it helps them to steady the camera. Tripods stands will be provided to participants by the research team upon request.

Patients can ask a partner, friend, or family member to take the photographs on their behalf if this is a possibility. Overall, the option will be up to the study participants.

Ideal lighting would be natural, with no bright glare and no shadows. However, this is hard to achieve without specific weather (such as overcast). It is recommended that participants should take two versions of a photograph: one with as best light as possible and the other using the phone’s flash. In addition, participants will be encouraged to record small videos (about 5 seconds) around the lesion.

On opening the app, the user presses the “Take a Picture” button and is immediately taken to the live camera view. The user should position the camera and their finger lesion in such a way that the lesion is in clear focus, central to the frame, and can be held in a steady position. The user can then press the shutter button to take the image. Once the image is captured it can be accepted and sent to the appropriate approved storage or it can be retaken if the participant is unhappy with the picture preview shown.

For recording video, a similar process is taken. The user presses the “Take a Video” button and is taken to the video capture page, where videos can be recorded. The video should be captured from as many angles as possible, while keeping the finger lesion central and in focus for the whole time.

The participant repeats the process for each finger lesion they wish to include in the study.

**Potentially identifiable characteristics revealed from app images / video recordings:** Participants will be advised to avoid collecting information that could potentially identify them when photographing or recording images of their finger lesions. To reduce this possibility the research team will advise participants not to talk whilst recording their lesion images (to avoid the possibility of voice recognition or mentioning identifying characteristics, names, addresses). They will also advise participants to clear the image background of identifiable information such as family photographs or mirrors that could reflect facial images. Additionally, if patients have any identifiable markings close to the lesion, such as tattoos or birthmarks, these should not be included in the image frame if possible. If images are sent to the research team with identifiable features, these will be blocked out / obscured by the research team and the patients will be contacted and advised of this for future reference.
