# Supplementary material for: Feasibility study of a smartphone app to monitor systemic sclerosis-related digital ulcers—a potential new tool for remote clinical monitoring
Source: Rheumatol Adv Pract. 2026 Mar 18;10(2):rkag035. doi: 10.1093/rap/rkag035 (PMC13070691; doi:10.1093/rap/rkag035)
Supplement: rkag035_Supplementary_Data [file rkag035_supplementary_data.zip › PNew_End_of_study_questionnaire_Supplementary.docx]

**
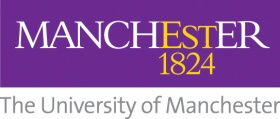
Development of a measuring app for finger lesions as an outcome measure for systemic sclerosis-related digital ulceration – Study 3**

**End of Study 3 – Feedback Questionnaire**

The following questionnaire asks about your experiences of using the smartphone app to image your finger ulcers and how useful did you find the app feedback relating to your ulcer healing rate

Patient ID:

**Following the imaging instructions**

For questions 1 to 7, please use the scales below to describe your experience of taking pictures/videos of your finger ulcers. In each case, circle the number that best describes your experience.

1. Remembering to take photographs/videos of finger ulcers on each occasion (twice per week)?

| **1** | **2** | **3** | **4** | **5** | **6** | **7** | **8** | **9** | **10** |
| --- | --- | --- | --- | --- | --- | --- | --- | --- | --- |
| **Very easy** |  |  |  |  |  |  |  |  | **Very difficult** |

1. Taking photographs/videos at the same time on each occasion (twice per week) ?

| **1** | **2** | **3** | **4** | **5** | **6** | **7** | **8** | **9** | **10** |
| --- | --- | --- | --- | --- | --- | --- | --- | --- | --- |
| **Very easy** |  |  |  |  |  |  |  |  | **Very difficult** |

1. Taking photographs/videos in the same place on each occasion (twice per week)?

| **1** | **2** | **3** | **4** | **5** | **6** | **7** | **8** | **9** | **10** |
| --- | --- | --- | --- | --- | --- | --- | --- | --- | --- |
| **Very easy** |  |  |  |  |  |  |  |  | **Very difficult** |

1. Keeping the environment and lighting the same on each occasion (twice per week) ?

| **1** | **2** | **3** | **4** | **5** | **6** | **7** | **8** | **9** | **10** |
| --- | --- | --- | --- | --- | --- | --- | --- | --- | --- |
| **Very easy** |  |  |  |  |  |  |  |  | **Very difficult** |

1. Making sure hands were in the same condition (e.g., no recent hand cream or hand washing) on each occasion (twice per week)?

| **1** | **2** | **3** | **4** | **5** | **6** | **7** | **8** | **9** | **10** |
| --- | --- | --- | --- | --- | --- | --- | --- | --- | --- |
| **Very easy** |  |  |  |  |  |  |  |  | **Very difficult** |

1. Overall experience of using the mobile phone to photograph/video your finger ulcer?

| **1** | **2** | **3** | **4** | **5** | **6** | **7** | **8** | **9** | **10** |
| --- | --- | --- | --- | --- | --- | --- | --- | --- | --- |
| **Very easy** |  |  |  |  |  |  |  |  | **Very difficult** |

1. Do you have any other comments about the imaging instructions used in this study? Please do not include any identifiable data such as your name when completing the comments sections.

**Taking Photographs**

For questions 8 to 11, please use the scales below to describe your experience of physically taking the photographs with your mobile phone. In each case, circle the item or number that best describes your experience.

1. Did you mainly hold the phone while taking photographs, did you place it on a surface and use the front-facing camera, or did someone lese take the photographs for you?

| Held the phone | Placed on surface | Someone else helped me |
| --- | --- | --- |

1. Holding the phone while imaging?

| **1** | **2** | **3** | **4** | **5** | **6** | **7** | **8** | **9** | **10** |
| --- | --- | --- | --- | --- | --- | --- | --- | --- | --- |
| **Very easy** |  |  |  |  |  |  |  |  | **Very difficult** |

1. Pressing the button or screen to take an image?

| **1** | **2** | **3** | **4** | **5** | **6** | **7** | **8** | **9** | **10** |
| --- | --- | --- | --- | --- | --- | --- | --- | --- | --- |
| **Very easy** |  |  |  |  |  |  |  |  | **Very difficult** |

1. Getting a good clear image of your finger ulcer?

| **1** | **2** | **3** | **4** | **5** | **6** | **7** | **8** | **9** | **10** |
| --- | --- | --- | --- | --- | --- | --- | --- | --- | --- |
| **Very easy** |  |  |  |  |  |  |  |  | **Very difficult** |

1. Do you have any other comments about the physical or practical aspects of taking the photographs? Please do not include identifiable data such as names

**Taking Videos**

For questions 13 to 16, please use the scales below to describe your experience of physically taking the photographs with your mobile phone. In each case, circle the item or number that best describes your experience.

1. Did you mainly hold the phone while taking videos, did you place it on a surface and use the front-facing camera, or did someone else take the videos for you?

| Held the phone | Placed on surface | Someone else helped me |
| --- | --- | --- |

1. Holding the phone while recording?

| **1** | **2** | **3** | **4** | **5** | **6** | **7** | **8** | **9** | **10** |
| --- | --- | --- | --- | --- | --- | --- | --- | --- | --- |
| **Very easy** |  |  |  |  |  |  |  |  | **Very difficult** |

1. Pressing the button or screen to start and stop capturing a video?

| **1** | **2** | **3** | **4** | **5** | **6** | **7** | **8** | **9** | **10** |
| --- | --- | --- | --- | --- | --- | --- | --- | --- | --- |
| **Very easy** |  |  |  |  |  |  |  |  | **Very difficult** |

1. Getting a good clear image of your finger ulcer?

| **1** | **2** | **3** | **4** | **5** | **6** | **7** | **8** | **9** | **10** |
| --- | --- | --- | --- | --- | --- | --- | --- | --- | --- |
| **Very easy** |  |  |  |  |  |  |  |  | **Very difficult** |

1. Do you have any other comments about the physical or practical aspects of taking videos? Please do not include identifiable data such as names

**App feedback**

For questions 18 to 20, please use the scales below to describe your experience of the app feedback you received relating to ulcer size, colour, and healing? In each case, circle the number that best describes your experience.

1. Was it helpful to know (via app feedback) whether your ulcer was improving (getting smaller) or worsening (getting bigger) over time?

| **1** | **2** | **3** | **4** | **5** | **6** | **7** | **8** | **9** | **10** |
| --- | --- | --- | --- | --- | --- | --- | --- | --- | --- |
| **Very helpful** |  |  |  |  |  |  |  |  | **Not helpful** |

1. Was it easy to understand the feedback provided via the app (was it presented in a clear and concise manner)?

| **1** | **2** | **3** | **4** | **5** | **6** | **7** | **8** | **9** | **10** |
| --- | --- | --- | --- | --- | --- | --- | --- | --- | --- |
| **Very easy** |  |  |  |  |  |  |  |  | **Very difficult** |

1. Would you be keen to use the app again (to monitor the progress of any further ulcers)?

| **1** | **2** | **3** | **4** | **5** | **6** | **7** | **8** | **9** | **10** |
| --- | --- | --- | --- | --- | --- | --- | --- | --- | --- |
| **Very keen** |  |  |  |  |  |  |  |  | **Not keen** |

1. As a result of the feedback you received via the app regarding your ulcer healing rate (was it improving, was it getting worse), were you less likely or more likely to seek additional advice from your consultant or specialist nurse?

| Less Likely to seek additional advice | 🗆 |
| --- | --- |
| More likely to seek additional advice | 🗆 |
| Neither more likely nor less likely | 🗆 |

1. Do you have any other comments about the app feedback? Please do not include identifiable data such as names

**General questions**

1. Having completed the study, do you think being asked to photograph/video your finger ulcer twice a week is: *(please tick one)*

| Too often (prefer once a week or less) | 🗆 |
| --- | --- |
| Not enough (prefer to take daily so it becomes part of my daily routine) | 🗆 |
| Twice weekly was fine for me! | 🗆 |

1. How easy was it to send your images/videos to the research team through the SALVE app? *(please circle)*

| **1** | **2** | **3** | **4** | **5** | **6** | **7** | **8** | **9** | **10** |
| --- | --- | --- | --- | --- | --- | --- | --- | --- | --- |
| **Very easy** |  |  |  |  |  |  |  |  | **Very difficult** |

1. Are there any additional features you would like to see in the app in the future? Please do not include identifiable data such as names
